# Osteoporosis is a Predictive Factor for Nephrolithiasis in an Adult Free-Living Caucasian Population From Southern Italy: A Longitudinal Retrospective Study Based on a General Practice Database

**DOI:** 10.1007/s00223-020-00737-9

**Published:** 2020-08-01

**Authors:** Domenico Rendina, Lanfranco D’Elia, Marco Evangelista, Gianpaolo De Filippo, Alfonso Giaquinto, Biagio Barone, Gaetano Piccinocchi, Domenico Prezioso, Pasquale Strazzullo

**Affiliations:** 1 Department of Clinical Medicine and Surgery, Federico II University, Via Sergio Pansini 5, 80131 Naples, Italy; 2 Hôpital Robert Debré, Paris, France; 3 Department of Neuroscience Reproductive Sciences and Dentistry, Federico II University, Naples, Italy; 4 “COMEGEN” Medical Cooperative, Naples, Italy

**Keywords:** Osteoporosis, Nephrolithiasis, Epidemiological survey

## Abstract

Osteoporosis and nephrolithiasis are common multifactorial disorders with high incidence and prevalence in the adult population worldwide. Both are associated with high morbidity and mortality if not correctly diagnosed and accurately treated. Nephrolithiasis is considered a risk factor for reduced bone mineral density. Aim of this retrospective longitudinal study was to evaluate if osteoporosis is a predictive factor for the nephrolithiasis occurrence. Free-living subjects referring to “COMEGEN” general practitioners cooperative operating in Naples, Southern Italy. Twelve thousand seven hundred ninety-four Caucasian subjects (12,165 female) who performed bone mineral density by dual-energy X-ray absorptiometry and have a negative personal history for nephrolithiasis. Subjects aged less than 40 years or with signs or symptoms suggestive of secondary osteoporosis were excluded from the study. In a mean lapse of time of 19.5 months, 516 subjects had an incident episode of nephrolithiasis. Subjects with osteoporosis had an increased risk of nephrolithiasis than subjects without osteoporosis (Hazard Ratio = 1.33, 95% Confidence Interval 1.01–1.74, *p* = 0.04). Free-living adult subjects over the age of 40 with idiopathic osteoporosis have an increased risk of incident nephrolithiasis, suggesting the advisability of appropriate investigation and treatment of the metabolic alterations predisposing to nephrolithiasis in patients with osteoporosis. The study protocol was approved by the ASL Napoli 1 Ethical Committee, protocol number 0018508/2018

## Introduction

Osteoporosis and nephrolithiasis are two common multifactorial disorders characterized by high incidence and prevalence in the adult population worldwide [1]. Both show high morbidity and mortality if not correctly diagnosed and accurately treated [1]. Osteoporosis is defined as a decrease in bone density that results in micro-architecture deterioration, which predisposes affected patients to fractures [2]. It is recognized as the most common form of metabolic bone disease, with an estimated 200 million people affected worldwide [3]. Approximately 30% of all postmenopausal women have osteoporosis in the USA and in Europe. At least 40% of these women and 15–30% of men will sustain one or more fragility fractures in their remaining lifetime [2]. Ageing of populations worldwide will be responsible for a major increase in the incidence of osteoporosis, particularly amongst postmenopausal women. The Italian National Health Institute (Istituto Superiore di Sanità, ISS) estimates that the prevalence of osteoporosis and osteopenia in the Italian population older than 40 years is 14.5% and 34.5% in males and 22.8% and 42.3% in females, respectively [4]. Symptomatic fractures occur in 21/1000 osteoporotic subjects per year with an estimated cost of 3 billion euro per year. Less than 20% of patients with symptomatic fractures reach a complete *restitutio* *ad* *integrum* [2]. Nephrolithiasis refers to the presence of crystalline stones (calculi) within the urinary system (kidneys and ureters) [5]. It affects nearly 1 in 11 individuals in the USA at some point in their lives, and there is evidence that the number of those who have had a stone is rising [5]. It has been estimated that more than 5% of Italian population over 35 years have already had an episode of kidney stones, and that kidney stones required more than 100,000 hospitalizations per year, with a cost that exceeds 200,000 Euro millions per year [6]. Similar epidemiological data are available in almost all industrialised countries [7]. Other than epidemiological data, both osteoporosis and nephrolithiasis share common pathogenic factors, such as unhealthy eating habits involving high salt, high sugar and inadequate calcium intakes as well as low physical activity and disorders of calcium–phosphate and vitamin D homeostasis [8–12]. Nephrolithiasis is considered a risk factor for reduced bone mineral density and osteoporosis [13], but data evaluating if osteoporosis predisposes to the occurrence of nephrolithiasis are lacking. Aim of this study was to assess if osteoporosis is a predictive factor for the occurrence of nephrolithiasis via a retrospective longitudinal study. The study was performed in free-living Caucasian adult subjects afferent to the Local Health Unit (Azienda Sanitaria Locale, ASL) Naples 1, Campania, Southern Italy.

## Methods

The study was based on the clinical records of 180,724 patients followed by the general practitioners (GPs) afferent to the “COMEGEN” (COoperativa di MEdicinaGENerale) Medical Cooperative operating within the ASL Naples 1. As of June 1st 2018, the participating GPs selected amongst their patients those who performed an evaluation of bone mineral density (BMD) by dual-energy X-ray absorptiometry (DXA, ICD9 code 8898) within December 1st 2017, according to Italian Ministerial Decree regulating Essential Assistance Levels (EAL) in osteoporosis management [14]. From the patients’ medical records, we collected data regarding weight, height, body mass index (BMI), age, sex (M = male, F = female), smoking habits (current, previous or never smoker [15], date of DXA, occurrence of nephrolithiasis (ICD9 codes 5920 to 5929) and relative date, and any pharmacological treatment for osteoporosis (i.e. single or combined use of calcium salts, vitamin D and analogues, selective estrogenic receptor modulators, bisphosphonates, teriparatide, strontium ranelate and/or denosumab) via a predefined data collection form. The selected patients were univocally identified with a sequential alphanumeric code, and their data were collected in an electronic file, elaborated by D.R., G.D.F. and M.E. Informed consent was obtained from all individual participants included in the study. The study protocol was approved by the Ethical Committee of ASL Napoli 1, protocol number 0018508/2018.

### Diagnostic Criteria

The diagnosis of osteoporosis was based on a T-score value measured by DXA ≤ − 2.5 in the lumbar spine, total hip or femoral neck, according to the World Health Organization (WHO) diagnostic criteria [16]. Subjects with a lumbar or femoral T-score > − 2.5 and a personal history negative for assumption of antiresorptive drugs were used as controls. The diagnosis of nephrolithiasis was based on the instrumental evidence of kidney stones and/or personal history of kidney stone ejection.

### Exclusion Criteria

A patient was excluded if the diagnosis of nephrolithiasis was antecedent the DXA date. Other exclusion criteria were: age lower than 40 years, malabsorption syndromes (ICD9 codes 5793 to 5799), rheumatoid arthritis (ICD9 code 7140), long-term immobilization, moderate to severe chronic kidney disease (estimated glomerular filtration rate < 60 ml/min; ICD9 codes 5853 to 5859, 586 and 6393), hyperthyroidism (ICD9 codes 24200 to 24291), primary hyperparathyroidism (ICD9 codes 25200 to 25208), hypoparathyroidism (ICD9 code 2521), Cushing’s syndrome (ICD9 code 2550), chronic liver disease (ICD9 codes 5710 to 5719), prostate cancer (ICD9 codes 185, 2334, 2365), pituitary tumours (ICD9 codes 1943, 2273, 2370), surgical history of terminal ileal resection (ICD9 code 4562), gastrectomy or small bowel bypass (ICD9 codes 430 to 4499), orchiectomy (ICD9 codes 622 to 6242), eating disorders (ICD9 codes 3071 and 30750 to 30759), alcoholism (ICD9 codes 30390 to 30393), regular use of gonadotropin-releasing hormone agonist, glucocorticoids, anticonvulsants, heparin, vitamin A, cytotoxic agents and antiandrogens, incomplete data collection, and osteoporosis therapy not compliant to Italian Medicine Agency (Agenzia Italiana del Farmaco, AIFA) prescriptive criteria [17].In this document, the AIFA prescribes “to guarantee before and during anti-osteoporotic treatment an adequate intake of calcium and vitamin D, resorting, if diet and sun exposure are inadequate, to supplements with calcium salts and vitamin D3 (not to its hydroxylated metabolites)” [17].

### Statistical Analysis

All statistical analyses were performed using the IBM SPSS Statistics software, version 23 (International Business Machines Corporation (IBM), Armonk, New York) by L.D.E, who did not participate to the data extraction from “COMEGEN” Medical Cooperative database. Analysis of variance (ANOVA) or Chi-squared test were used to assess differences in baseline main characteristics. Cox proportional hazard models were used to estimate hazard ratios (HRs)—with 95% confidence intervals (CIs)—for the risk of nephrolithiasis incidence in subjects with osteoporosis. The multivariate model was adjusted for age, sex, BMI, smoking habits, and treatment for osteoporosis. Subjects who did not develop nephrolithiasis were censored at data extraction. All reported *p* values are two-sided, and the significant level was set at *p* < 0.05.

## Results

**Fig. 1:**
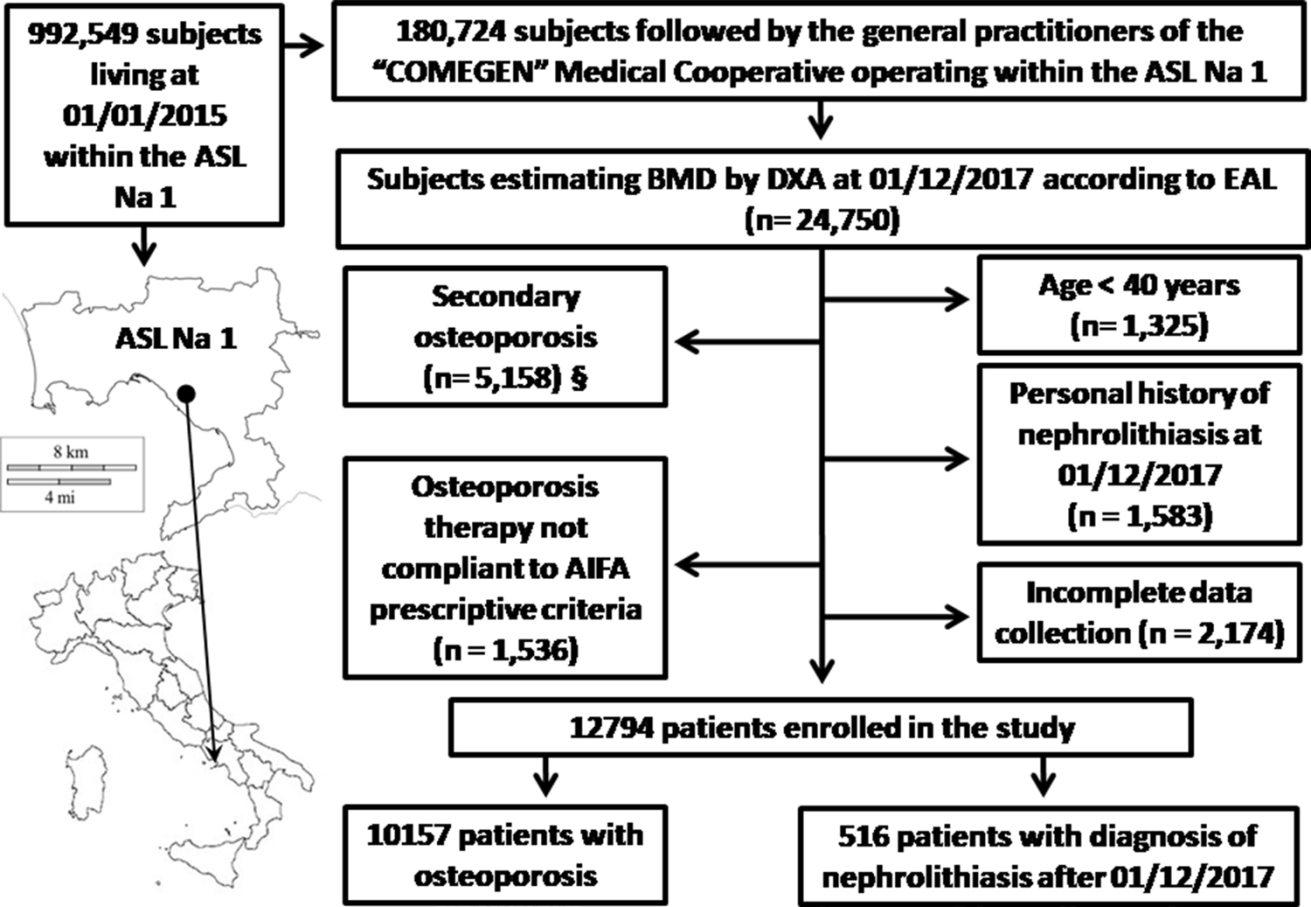
Study flow chart. ASL Na 1: Local Health Unit (in Italian: Azienda Sanitaria Locale) Naples 1. BMD: Bone mineral density. DXA: Dual-Energy X-ray absorptiometry. EAL: Essential Assistance Levels in osteoporosis management [14]. AIFA: Italian Medicine Agency (in Italian: Agenzia Italiana del FArmaco) [17].§: Subject with malabsorption syndromes (ICD9 codes 5793 to 5799), rheumatoid arthritis (ICD9 code 7140), long-term immobilization, moderate to severe chronic kidney disease (ICD9 codes 5853 to 5859, 586 and 6393), hyperthyroidism (ICD9 codes 24200 to 24291), primary hyperparathyroidism (ICD9 codes 25200 to 25208), hypoparathyroidism (ICD9 code 2521), Cushing’s syndrome (ICD9 code 2550), chronic liver disease (ICD9 codes 5710 to 5719), prostate cancer (ICD9 codes 185, 2334, 2365), pituitary tumours (ICD9 codes 1943, 2273, 2370), surgical history of terminal ileal resection (ICD9 code 4562), gastrectomy or small bowel bypass (ICD9 codes 430 to 4499), orchiectomy (ICD9 codes 622 to 6242), eating disorders (ICD9 codes 3071 and 30750 to 30759), alcoholism (ICD9 codes 30390 to 30393), regular use of gonadotropin-releasing hormone agonist, glucocorticoids, anticonvulsants, heparin, vitamin A, cytotoxic agents and antiandrogens, were excluded from the study

According to the inclusion and exclusion criteria (Fig. 1), we selected the medical records of 12,794 patients (12,165 F: 629 M; mean age 69.3 ± 10.0 years; BMI 26.8 ± 4.8 kg/m^2^): 10,157 subjects (9758 F: 399 M; 70.0 ± 9.7 years; BMI 26.7 ± 4.7 kg/m^2^) have clinical diagnosis of osteoporosis whereas the remaining 2637 subjects (2407 F: 230 M; mean age 66.6 ± 10.7 years; BMI 27.2 ± 5.0 kg/m^2^), which have a BMD > − 2.5 T-score, were considered as controls. All of them have a personal history negative for nephrolithiasis at the time of DXA execution.

**Fig. 2:**
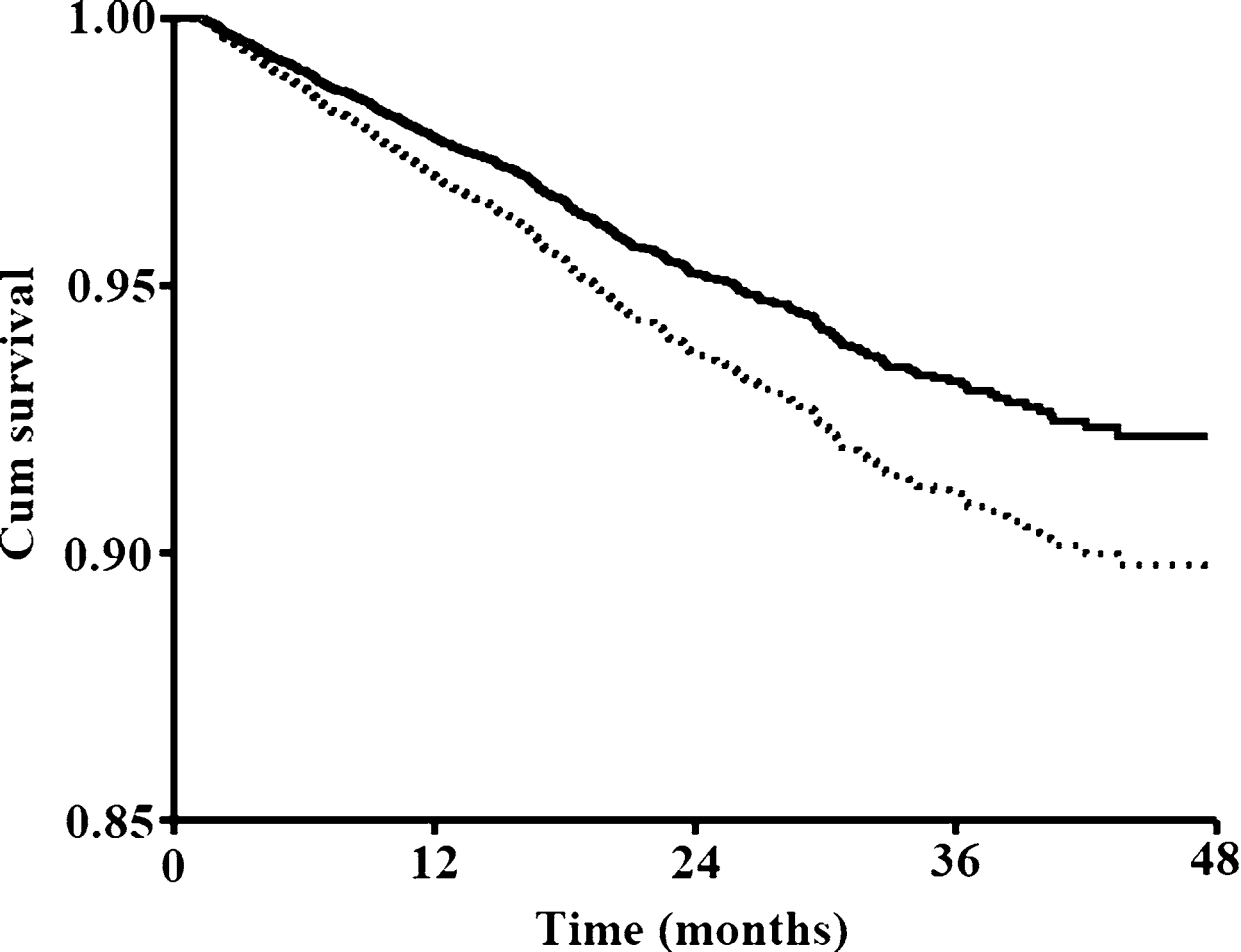
Time-dependent likelihood of the occurrence of nephrolithiasis. Dotted line and continuous line denote patients with or without osteoporosis at baseline, respectively

In a mean lapse of time of 19.5 ± 14.4 months (minimum: 1.5 months; maximum: 113.7 months, median: 15.9 months, person-months: 249,554, rate: 2.1 cases × 1000 person-months), 516 subjects had clinical diagnosis of nephrolithiasis occurred after the execution of the DXA (4%; 494 F: 22 M; mean age 70.8 ± 8.8 years; BMI 26.6 ± 4.8 kg/m^2^; 447 with osteoporosis; average nephrolithiasis onset: 15.4 ± 11.2 months). The percentage incidence of nephrolithiasis in subjects divided according to age range was 2.1%, 2.2%, 4.3%, 5.0% and 4.0% in subjects aged between 40 and 50 years (*n* = 329), between 51 and 60 years (*n* = 2385), between 61 and 70 years (*n* = 4169), between 71 and 80 years (*n* = 1752) and > 81 years (*n* = 1752), respectively (*p* = 0.001). The percentage number of subject developing nephrolithiasis was higher amongst subjects with osteoporosis compared with patients without osteoporosis (4.4% vs 2.6%, for subjects with and without osteoporosis respectively; *p* < 0.001). At the univariate Cox model, osteoporosis diagnosis at baseline was associated with an increased risk of nephrolithiasis (HR = 1.33, 95% CI 1.03–1.71, *p* = 0.03). This association remained significant in a model adjusted for age, sex, BMI, treatment for osteoporosis and smoking habits (HR = 1.33, 95% CI 1.01–1.74, *p* = 0.04; Table 1) as well as analysing only subject receiving the clinical diagnosis of nephrolithiasis at least 6 months after the DXA execution (*n* = 394, 3.8%; 375 *F*: 19 M; mean age 71.6 ± 8.7 years; BMI 26.8 ± 4.7 kg/m^2^, mean follow up time: 23.3 months, HR = 1.50, 95% CI 1.08–2.09, *p* = 0.01). The time-dependent likelihood of the occurrence of nephrolithiasis in patients with or without osteoporosis was reported in Fig. 2.

**Table 1:** Risk factors for incidence of nephrolithiasis in free-living subjects from southern Italy over 40 years old

	*p*	HR	95% CI	
Osteoporosis diagnosis (y/n)	0.04	1.33	1.01	1.74
Age (years)	0.02	0.99	0.98	1.00
Sex (m/f)	0.52	0.87	0.56	1.34
BMI (Kg/m^2^)	0.22	0.99	0.97	1.01
Osteoporosis treatment (y/n)	0.39	1.15	0.84	1.56
Current smokers (y/n)	0.18	1.17	0.93	1.48
Previous smokers (y/n)	0.68	1.08	0.73	1.60

Osteoporosis diagnosis was rendered according to the World Health Organization (WHO) diagnostic criteria: T-score value measured by Dual-Energy X-ray absorptiometry ≤ − 2.5 in the lumbar spine, total hip or femoral neck [14]. HR: hazard ratio. CI: confidence interval. BMI: body mass index. Osteoporosis treatment: current or previous single or combinate use of calcium salts, vitamin D and analogues, selective estrogenic receptor modulators, bisphosphonates, teriparatide, strontium ranelate, or denosumab

*Y*/*n* yes or not. *M*/*f* male or female

## Discussion

The results of our study demonstrate that in free-living adult subjects over 40 years old without personal history of nephrolithiasis, idiopathic osteoporosis is a predictive factor for the incidence of nephrolithiasis. The predictive value of osteoporosis remains after correction for sex, BMI, age, smoking habits and treatment for osteoporosis. The relevance of study results is emphasized by the high prevalence of both disorders in the general population and the high rate of nephrolithiasis incidence after the age of 40 [1]. A recent meta-analysis, including 28 case-controls studies (1595 patients with nephrolithiasis compared to 3402 healthy controls), demonstrated that nephrolithiasis is associated with lower BMD and increased risk of osteoporosis and fractures [13]. A subsequent prospective study confirmed that nephrolithiasis could be considered a risk factor for reduced BMD and occurrence of osteoporosis [18]. To our best knowledge, however, no data are available in the international literature indicating that osteoporosis is a predictive factor for the incidence of nephrolithiasis in the adult population. In accordance with our results, Prochaska et al. demonstrated in the Nurses’ Health Study II population that a reduced bone density was an independent risk factor for incident kidney stone and that the use of bisphosphonate was associated with lower risk of incident kidney stones [19]. Some data suggest a pathogenic relationship between osteoporosis and nephrolithiasis. Calcium intake is one of the many factors affecting the development of peak bone mass and preservation of bone mass in adults [20]. Epidemiological studies demonstrate a progressive reduction in dietary calcium intake of subjects with increasing age, resulting in a negative calcium balance [21, 22]. This mechanism, in turn, significantly increases the risk of osteoporosis and calcium nephrolithiasis over time. Calcium dietary intake is a key factor for oxalate absorption and excretion, which is one of the most potent promoters of calcium nephrolithiasis [23]. The reduction of calcium dietary intake decreases the calcium concentration in the intestinal lumen and prevents the formation of insoluble and non-absorbable calcium–oxalate salts. In this way, a higher quantity of soluble oxalate salts remains free in the intestinal lumen and can be easily absorbed and excreted by kidney, increasing urinary oxalate excretion, a significant metabolic risk factor for nephrolithiasis [24, 25]. In addition, considering that the follicular stimulating hormone is known to have a stimulatory effect on the osteoclast activity, its increase after menopause and andropause could also play a significant role in the pathogenesis of both nephrolithiasis and osteoporosis, increasing bone loss and urinary calcium excretion [26, 27]. Finally, experimental studies suggest a possible relationship between hypocitraturia, a recognized metabolic risk factor for calcium nephrolithiasis, and reduced BMD and osteoporosis [28, 29]. For this study we used an administrative database. According to Johnson et al., this study methodology shows advantages and limits [30]. In this regard, it should be noted that the definition of osteoporosis based upon administrative health database achieves an acceptable level of sensitivity, specificity, and accuracy, in particular when the assumption of anti-osteoporotic drugs was used as additional information in the query formulation, as in our study [31]. In addition, the use of ICD-9 coding for urinary calculi is sufficiently valid to be useful in studies using administrative data to analyze nephrolithiasis incidence [32]. Additional strengths of our study are the high sample size and the use of stringent diagnostic criteria that permitted the recruitment of subjects with or without idiopathic osteoporosis and nephrolithiasis. The most relevant and common causes of secondary osteoporosis and nephrolithiasis were excluded. Moreover, both the diagnosis of osteoporosis and that of nephrolithiasis were performed by qualified physicians not involved in the statistical analysis of collected data using strong and objective clinical criteria. Finally, the control subjects were selected based on DXA T-score >− 2.5 and thus were not osteoporotic, according to WHO diagnostic criteria for osteoporosis. Some limits of the study are also evident. This is not a prospective study *stricto* sensu. We are also unable (a) to perform a “dose effect” analysis to observe if the grade of reduction of BMD, evaluated by MOC-DXA, is associated with a higher risk of nephrolithiasis; (b) to evaluate if the anatomical site of DXA exam furnishing pathological data (i.e. lumbar, femoral or ulnar site) influences the study results; (c) to assess the type of renal calculi occurring in patients with osteoporosis; (d) to evaluate the role of metabolic risk factor for both osteoporosis and nephrolithiasis, i.e. hyperoxaluria, hypocitraturia and hypercalciuria, in the pathogenesis of the observed association; and (e) to perform a multivariate analysis which take into account the dietary habits of study cohorts. It is finally possible that episodes of asymptomatic nephrolithiasis linked to stones as small as grain of sand were not reported in the database. All these limits are principally linked to kind of data used in this study which is based on administrative data [30]. We also excluded from the study subjects with a moderate to severe chronic kidney disease (CKD), considering that these patients have an increased risk of developing disorders of bone and mineral metabolism, i.e. CKD-mineral and bone disorder (CKD–MBD), requiring an integrated approach [33]. We then limited the analysis to subjects with low risk of CKD. Further studies, using different methodologies, are needful to clarify the possible influence of aforementioned factors, in particular dietary habits and values of GFR identifying subjects at low risk of CDK, in the pathogenesis of increased risk for nephrolithiasis observed in subjects with osteoporosis. In the examined cohort there was a large majority of female subjects (more than 95%); however, it should be noted that this is the first study that analysed both disorders in more than 400 male subjects over 40 years old. Based on our study results, patients with osteoporosis should be evaluated for nephrolithiasis occurrence. We suggest for patients with idiopathic osteoporosis to perform an abdomen ultrasonography and a complete evaluation of metabolic risk factors for nephrolithiasis (i.e. urinary excretion of calcium, oxalate, citrate, magnesium, phosphate and urate) within 1 year from osteoporosis diagnosis and later on at least once a year. Considering the distribution of nephrolithiasis incidence in subjects with different age ranges, this approach appears particularly indicated in subjects over the age of 61. Regarding possible medical interventions for a correct primary prevention of nephrolithiasis in patients with idiopathic osteoporosis, it should be noted that the non-pharmacologic interventions advised for the prevention and management of osteoporosis and nephrolithiasis are similar. Both include avoidance of weight gain or reduction of overweight, avoidance of smoking and alcohol abuse and regular practice of physical activity [34]. In addition, for prevention of cardiovascular disease, osteoporosis and nephrolithiasis, a normal calcium (1.2–1.5 g a day) low sodium diet (< 5 g of salt a day), with large consumption of vegetable proteins and a water intake of at least 2 L per day are also recommended [10, 35–37]. According to Ferraro et al. [35], this lifestyle and nutritional approach reduces by over 60% the risk of nephrolithiasis. Regarding pharmacological treatment of osteoporosis and nephrolithiasis, considering the role of citrate in the pathogenesis of both disorders, we propose they will be carried out ad hoc clinical trials should be performed to evaluate the use of this alkalinizing agents for treatment of both these strictly related disorders [38, 39]. In conclusion, the study results demonstrate that free-living adult subjects over the age of 40 with idiopathic osteoporosis have an increased risk of incident nephrolithiasis, suggesting the advisability of appropriate investigation and treatment of the metabolic alterations predisposing to nephrolithiasis in patients with osteoporosis.
